# Divergent effects of repeated cocaine and novel environment exposure on locus coeruleus *c‐fos* expression and brain catecholamine concentrations in rats

**DOI:** 10.1002/brb3.1222

**Published:** 2019-02-20

**Authors:** Michael J. Lisieski, Klevis Karavidha, Ali Gheidi, Rafael L. Garibyan, Alana C. Conti, Jonathan D. Morrow, Shane A. Perrine

**Affiliations:** ^1^ Department of Psychiatry and Behavioral Neurosciences Wayne State University School of Medicine Detroit Michigan; ^2^ Department of Psychiatry University of Michigan Medical School Ann Arbor Michigan; ^3^ Research and Development Service, John D. Dingell VA Medical Center, Department of Neurosurgery Wayne State University School of Medicine Detroit Michigan

**Keywords:** cocaine, locus Coeruleus, norepinephrine, novelty

## Abstract

**Introduction:**

Chronic administration of cocaine causes a disinhibited, hyperexploratory response to novel environments. As the norepinephrine (NE) system regulates exploration and is dysregulated following cocaine exposure, we hypothesized that this cocaine‐mediated hyperexploratory response is associated with increased locus coeruleus (LC) reactivity.

**Methods:**

To test this hypothesis, we used dual fluorescent in situ hybridization immunofluorescence to analyze novelty‐induced *c‐fos* and tyrosine hydroxylase expression in the LC and high‐pressure liquid chromatography to measure dopamine (DA) and NE concentrations in key catecholamine projection regions following exposure to cocaine.

**Results:**

Repeated cocaine exposure followed by a 14‐day drug‐free period increased exploration of novel environments, replicating previous findings. Novelty exposure increased LC *c‐fos* expression, increased anterior cingulate NE, and decreased ventral tegmental area DA. Cocaine exposure decreased amygdala (AMY) DA, but had no effect on LC *c‐fos* expression or NE in any tested brain region. No interactions between cocaine and novelty were found. Open arm exploration was positively correlated with LC *c‐fos* expression and NE concentrations in both the anterior cingulate and nucleus accumbens, and negatively correlated with AMY DA concentration.

**Conclusions:**

Our findings confirm that exposure to novel environments increases LC activity and NE in the anterior cingulate cortex, that long‐term exposure to cocaine dysregulates AMY DA, and that disinhibited exploration in novel environments correlates with NE and DA in regions that modulate risk‐taking and avoidance behavior. Further studies investigating the effects of cocaine on brain catecholamine systems are important in understanding the long‐lasting effects of cocaine on brain function.

## 1 INTRODUCTION

Cocaine use is a major and growing public health problem. The number of individuals using cocaine in the United States has risen significantly since a recent low point in 2013 (Johnston, O'Malley, Bachman, Schulenberg, & Miech, 2016). Cocaine overdose has become more prevalent over the past decade (Hedegaard, Warner, & Miniño, 2017), with cocaine remaining the most common illicit drug involved in overdose deaths among black men and women in the United States (Shiels, Freedman, Thomas, & de Gonzalez, 2017). Furthermore, there are no approved pharmacotherapies for cocaine use disorder (Shorter, Domingo, & Kosten, 2015). The persistence and recent regrowth of cocaine use as a public health problem highlights the need for continued study of its effects on brain function.

Cocaine binds to and inhibits monoamine transporters, including dopamine (DA) and norepinephrine (NE) transporters (Ritz, Cone, & Kuhar, 1990). Cocaine‐induced neuroadaptations in dopaminergic neurons have been extensively studied and are instrumental in cocaine reward and cocaine‐cue associations (Volkow, Wise, & Baler, 2017), as well as craving and relapse to cocaine use (Wolf, 2016). The effects of cocaine on NE systems in the brain are less well‐studied, but appear to be involved in several clinically relevant aspects of cocaine‐induced behavioral and neuronal changes. NE regulates processes such acute withdrawal‐induced anxiety (Harris & Aston‐Jones, 1993), cocaine‐primed reinstatement of cocaine seeking (Schmidt et al., 2017), locomotor activity (LMA) sensitization (Drouin, Blanc, Villégier, Glowinski, & Tassin, 2002), reward (Drouin, Darracq et al., 2002), and cocaine‐induced DA release in the nucleus accumbens (NAC) (Carboni et al., 2001). While a sizable body of research has investigated how manipulations of the NE system affect behaviors related to drug addiction (Zaniewska, Filip, & Przegaliński, 2015), how the NE system itself adapts following repeated administration of cocaine remains unclear. There is, however, some evidence that NE systems are dysregulated following repeated cocaine administration; fetal (Elsworth et al., 2007) and adult (Pitts & Marwah, 1989) rats repeatedly exposed to cocaine show reduced α2‐adrenergic receptor mediated inhibition of NE release by the locus coeruleus (LC), the primary source of NE to the forebrain. Additionally, cocaine self‐administration upregulates the NE transporter in noradrenergic projection fields in monkeys (Beveridge, Smith, Nader, & Porrino, 2005), a finding that has also been observed in humans using postmortem brain tissue (Mash, Ouyang, Qin, & Pablo, 2005). These studies show that the NE system is dysregulated following cocaine exposure, but more specific investigation of cocaine‐induced changes in NE function is necessary in order to refine our understanding of the pathophysiology of cocaine use disorder and allow the rational development of therapeutics targeting this system.

In a previous study, we observed that rats treated with binge‐pattern cocaine followed by an extended drug‐free period exhibited a disinhibited, hyperexploratory phenotype (Lisieski & Perrine, 2017). Similar observations have been made by other investigators following cocaine self‐administration (Mantsch et al., 2008) and amphetamine injections (Olausson, Engel, & Söderpalm, 2000). These findings mirror behavioral abnormalities seen in rats bred for high reactivity in novel environments, which exhibit several addiction‐related traits including impulsivity and increased motivation to take cocaine (Flagel, Waselus, Clinton, Watson, & Akil, 2014). This high‐reactivity phenotype is associated with increased NE in the NAC and is dependent on noradrenergic neurotransmission (Mabrouk et al., 2017), indicating that abnormal NE signaling may be important in such risk‐conferring, disinhibited phenotypes which are phenomenologically similar to those we and others have observed following repeated exposure to cocaine and withdrawal from cocaine.

In this study, we hypothesized that exposure to novel environments would activate the LC more strongly in rats with a history of repeated cocaine exposure than in control rats, leading to increased NE in midbrain and forebrain regions that regulate exploratory behavior. To test this, we exposed rats to repeated binge‐pattern cocaine or saline and then, after a 14‐day drug‐free period, exposed them either to a series of novel environments or to routine handling in a fully crossed design. Following this, we determined the extent to which novelty exposure activated LC cells by quantifying expression of the immediate early response gene *c‐fos*, a marker of recent strong neuronal activity, using dual fluorescence in situ hybridization immunofluorescence (FISH‐IF). Finally, we measured DA and NE concentrations in the ventral tegmental area (VTA), amygdala (AMY), anterior cingulate cortex (ACC), and NAC using high‐pressure liquid chromatography (HPLC) to determine if previous exposure to binge‐pattern cocaine enhanced either baseline or novelty‐elicited catecholamine levels in these brain regions.

## 2 MATERIALS AND METHODS

**Figure 1:**
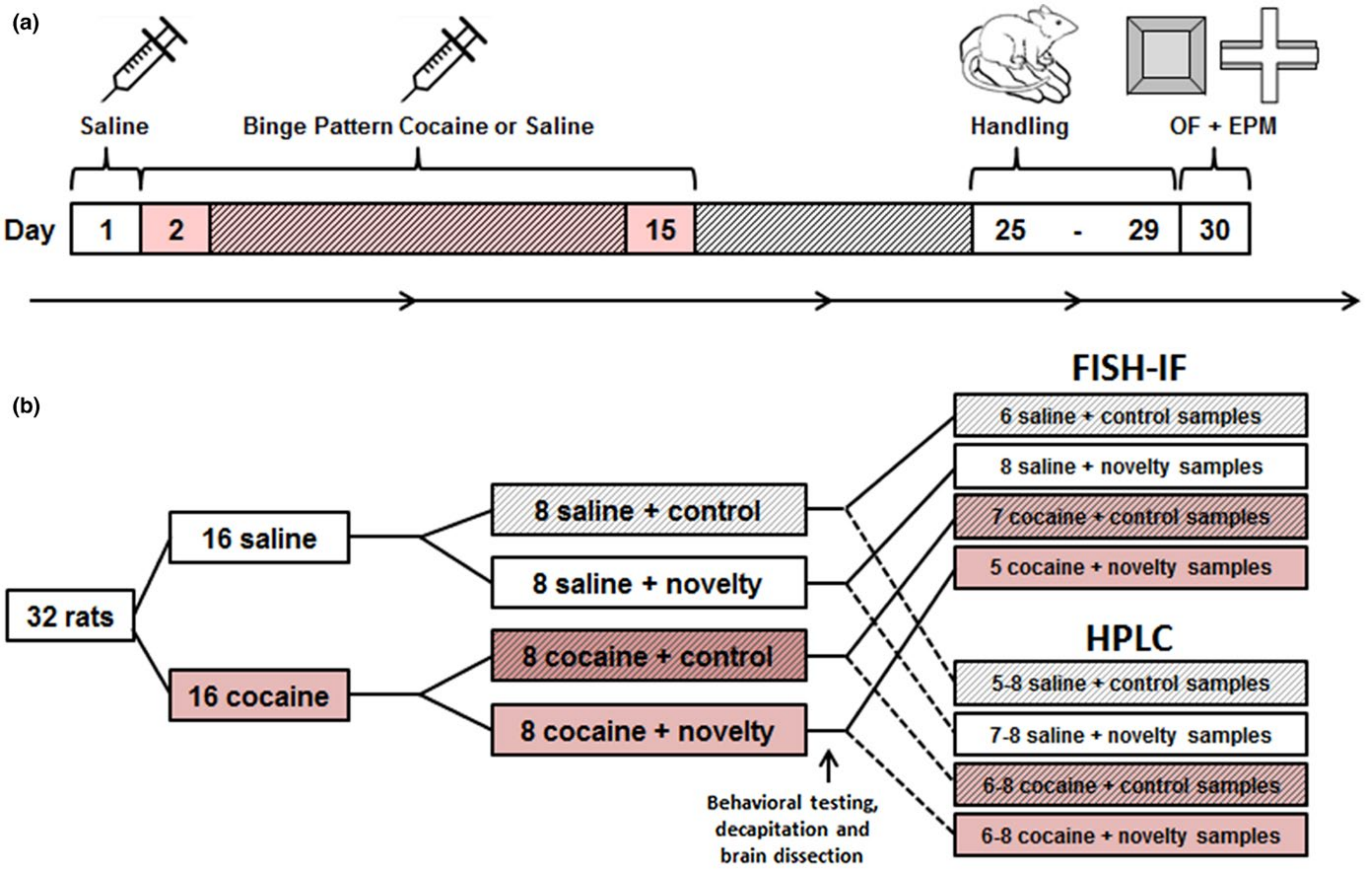
Timeline and group assignment. Rats were given 14 days (days 2–15) of binge pattern cocaine exposure (three injections of 15 mg/kg of cocaine per day, timed 1 hr apart) or injections of isovolumetric saline, then given a 14‐day drug‐free period (days 25–29) and subsequently exposed to either the open field (OF) and elevated plus maze (EPM) or routine handling on the final day of testing (day 30; a). Thirty‐two rats were used in total, with eight in each treatment group; some postmortem fluorescence in situ hybridization immunofluorescence (FISH‐IF) and high‐pressure liquid chromatography (HPLC) analyses included fewer samples (b)

An overview of the study design is shown in Figure 1a; group assignment and sample sizes are shown in Figure 1b. First, rats were treated for 14 days with thrice‐daily binge‐pattern cocaine or saline injections (Perrine, Schroeder, & Unterwald, 2005). After a 14‐day drug‐free period, rats were exposed to either a novelty (behavioral testing) or non‐novelty (transport and handling only) condition and then euthanized. Their brains were dissected, *c‐fos* mRNA expression in the LC was measured using a dual FISH‐IF protocol, and NE and DA concentrations in selected brain regions were analyzed using HPLC.

### 2.1 Subjects

In total, 32 male Sprague–Dawley rats (Charles River Laboratories, Wilmington, MA, Strain Code 400) weighing 225–275 g at the beginning of the study were used. Rats were pair‐housed upon arrival and acclimated to a climate‐controlled vivarium under a reverse light/dark cycle (lights off at 600, lights on at 1800) for 1 week. Rats were given ad libitum access to food and water except during behavioral testing. All procedures were conducted during the dark phase under dim red light. Cage mates were always in the same experimental group. On each experimental day, rats were transported to the laboratory 1 hr prior to procedures. Procedures were approved by the WSU Institutional Animal Care and Use Committee and followed relevant guidelines in the NIH Guide for the Care and Use of Laboratory Animals. Rats were randomly assigned to experimental groups at the beginning of the study.

### 2.2 Binge pattern cocaine administration

To establish baseline locomotor activity (LMA), rats were given three intraperitoneal injections of sterile saline 1 hr apart in a LMA monitoring apparatus (Digiscan DMicro, Accuscan Instruments, Columbus, OH) consisting of a clear plastic cage within an array of 16 photobeam emitter/detector pairs. The number of beam breaks per minute was recorded 30 min prior to the first injection until 1 hr after the third injection. On each of the 14 days of binge‐pattern cocaine administration, rats were given three intraperitoneal injections, 1 hr apart, of either 15 mg/kg (‐) cocaine HCl (NIDA Drug Supply Program, Bethesda, MD) in saline (0.9% NaCl) or isovolumetric saline. On the first and last day of cocaine administration, injections were given in the LMA monitoring apparatus; on other days, injections were given in home cages. Following the last day of cocaine administration, rats were assigned to novelty or control conditions; groups were balanced for the amount of cocaine sensitization observed.

### 2.3 Behavioral testing

During the last 5 days of the drug‐free period, rats were habituated daily by brief handling (~30 s) in the testing room four times per day with 10 min between each handling session. On the day of behavioral testing, half of the rats in each drug group were given behavioral testing in novel environments (novelty condition), while the other half were briefly handled four times as on habituation days (control condition). Rats assigned to the novelty condition were exposed to the open field (OF) for 10 min, held in a novel round glass chamber for 10 min, and then exposed to the elevated plus maze (EPM) for 10 min.

#### 2.3.1 Open field

At the beginning of behavioral testing, each rat was placed into the corner of an open‐topped black Plexiglas box measuring 80 × 80 × 36 cm (Form Tech Plastics, Oak Park, MI) and behavior was recorded from overhead for 10 min. These recordings were analyzed using Ethovision XT 11 (Noldus Information Technology, Wageningen, The Netherlands) to determine the amount of time spent in the outer 50% of the apparatus (thigmotaxis), and total distance traveled.

#### 2.3.2 Elevated plus maze

Rats were tested in a black Plexiglas EPM apparatus (Coulbourn Instruments, Allentown, PA) with two open arms (with a 1 cm tall edge/lip) and two closed arms (with 30 cm tall side walls) elevated 52 cm off the ground. Each arm was 10 cm wide × 45 cm long. Behavior was recorded for 10 min and recordings were analyzed using Ethovision to quantify the number of arm entries, distance traveled, and time spent in the open arms.

### 2.4 Combined *c‐fos* fluorescent in situ hybridization and tyrosine hydroxylase (TH) immunofluorescence

Rats were rapidly decapitated without anesthesia 90 min after the beginning of behavioral testing or handling. This is within the time window following acute stress during which *c‐fos* expression in the LC is maximal (Cullinan, Herman, Battaglia, Akil, & Watson, 1995). Brains were immediately removed and flash‐frozen in isopentane cooled on dry ice, then stored at −80°C. Brains were coronally bisected rostral to the pons and the posterior portions were sectioned at 20 µm. Sections containing the LC (Bregma: −9.5 to –10.0 mm) (Paxinos & Watson, 2007) were mounted on glass slides. Sections underwent a combined FISH‐IF protocol for quantification of *c‐fos* mRNA and TH protein density within the LC.

FISH to label *c‐fos *mRNA was performed as previously described (Guzowski & Worley, 2001). Ribo‐probe against the *c‐fos* antisense strand was synthesized through in vitro transcription using DNA grown in plasmid vector (gifted by Dr. Stanley Watson) and transcripts were conjugated to digoxigenin. Sections containing the LC were incubated overnight with ribo‐probe at 56°C. Next day, the sections were washed 4x in sodium citrate buffer and incubated with anti‐digoxigenin antibody (1:400; Roche 11207733910). Ribo‐probe bound to *c‐fos* was visualized using Cy3 (1:50; Perkin Elmer NEL744001KT). Following confirmation of successful hybridization by visualization of FISH signal, brain sections were incubated in PBS for 5 min, incubated in blocking cocktail (1X PBS 0.2% triton × 2.6% BSA) for 1 hr, washed again, and incubated overnight with anti‐TH antibody (1:500; Abcam ab76442) at 4°C. Next day, the sections were washed with PBS and incubated for 4 hr with a secondary antibody conjugated to Alexa 488 (1:500; Abcam ab150169) in PBS. Sections were washed in PBS with 0.1% triton X, counterstained with DAPI (1:1,000; Sigma), coverslipped, sealed, and kept at 4°C until imaged.

### 2.5 Imaging and image analysis

Three‐channel images of the LC were collected using a Nikon Eclipse Ni‐E fluorescent microscope and NIS‐Elements AR 4.20.00. Image analysis was conducted using ImageJ (Schneider, Rasband, & Eliceiri, 2012). TH labeling was used to define the boundaries of the LC, and average density of TH and *c‐fos* signal within this region was quantified in all images in which the LC could be unambiguously identified. Background values were obtained by selecting four 100 × 100 pixel regions with no cellular‐patterned staining outside of the LC, calculating the density of signal within them, and averaging the three lowest values. When multiple sections were analyzed from a single animal, background‐corrected TH and *c‐fos* intensity values (LC value/background value) were averaged to yield a single background‐corrected value for that animal. Measurements were done by a scorer blind to experimental conditions. An analysis of inter‐rater reliability was conducted using measurements taken by a secondary scorer.

### 2.6 HPLC of NE and DA

The anterior portion of each brain was sectioned coronally into 2 mm sections using surgical‐grade razor blades and a chilled steel brain matrix. Tissue punches (1.5 mm in diameter) were taken from the NAC, ACC, AMY, and VTA (Paxinos & Watson, 2007). These tissue punches were stored at –80°C until HPLC analysis of whole‐tissue NE and DA levels.

Frozen tissue punches were weighed, sonically disrupted in 0.2 N HClO_4_, held at 4°C, and centrifuged at 14,000 *g* for 10 min. A 50 µl aliquot of supernatant from each sample was removed and analyzed with a Dionex Ultimate 3000 UHPLS system (Thermo Scientific, Waltham, MA). A 10 µl portion of each sample was injected at 4°C onto a C_18_‐RP column maintained at 25°C. TEST acetonitrile mobile phase (Thermo Scientific) was used. Coulometric detection was achieved with an ultra‐analytical dual electrode cell (Thermo Scientific) set at –175 mV (reference electrode) and 300 mV (working electrode). Gain for both electrodes was 100 µA. A guard cell (ESA guard cell model 5020) set to 350 mV and guard column (2.1/3.0 mm ID, Thermo Scientific) were used. Chromatograms were analyzed using Chromeleon 7 software (Dionex) to quantify peak height. Catecholamine concentrations were calculated (ng catecholamine/mg wet tissue weight; ng/mg) by comparison to external standards run in parallel with brain samples (Sigma‐Aldrich, St. Louis, MO). The detection threshold was set at three times the average baseline noise. Only samples with signal exceeding the detection threshold were used, yielding lower sample sizes in some HPLC results compared to *c‐fos* and behavioral outcomes.

### 2.7 Data analysis

Databasing and analysis were conducted using Excel 2016 (Microsoft) and SPSS 24/25 (IBM). Locomotor activity data were analyzed using independent samples *t* tests comparing saline‐ to cocaine‐exposed rats at each time point, and paired *t* tests to compare the activity of the cocaine‐exposed group between the first and last day of cocaine exposure. Data from the OF test and EPM were analyzed using two‐tailed independent samples *t* tests comparing cocaine‐ and saline‐treated rats. Background‐corrected measurements of LC *c‐fos* and TH and catecholamine data were analyzed using two‐way ANOVA, with cocaine and novelty exposure entered as between‐subject factors. No post hoc tests were conducted. Pearson's *R* was used to determine the inter‐rater reliability of FISH‐IF analyses, and to determine if LC *c‐fos* expression and catecholamine concentrations were significantly correlated with behavioral measures. These correlational analyses included both saline‐ and cocaine‐exposed rats, but only included the novelty‐exposed groups, as no behavioral data were collected from rats not exposed to the novel testing environments. For all analyses, α = 0.05.

## 3 RESULTS

### 3.1 Cocaine‐induced locomotor sensitization

**Figure 2:**
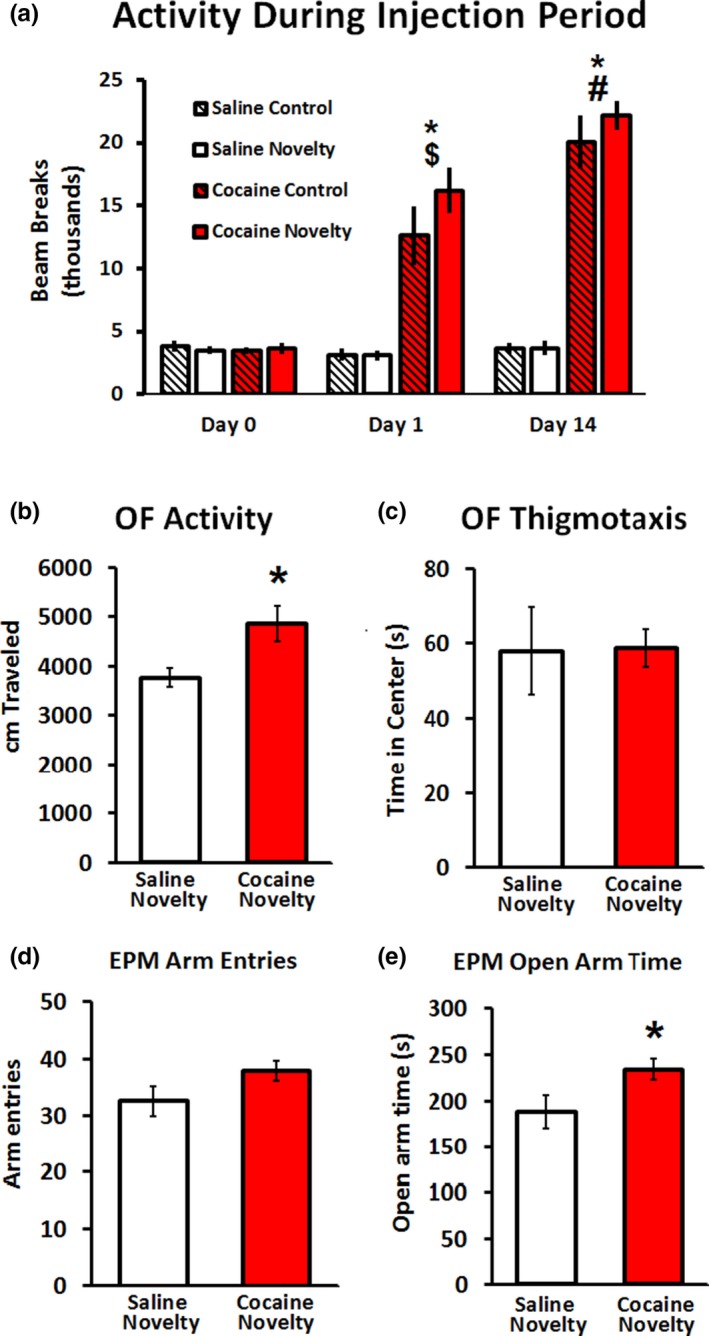
Cocaine exposure induced sensitization and a long‐lasting disinhibited phenotype. Treatment groups showed no differences in locomotor activity (LMA) at baseline (a). Rats given cocaine showed significantly more LMA than rats given saline on the first and last days of drug administration. Rats given cocaine had greater LMA on the last day compared to the first, indicating the cocaine‐induced sensitization occurred. While control and novelty groups are displayed separately on this graph to illustrate lack of difference in LMA and sensitization, statistical analyses were conducted by comparing all saline rats to all cocaine rats. Cocaine‐exposed rats showed greater LMA in open field (OF) than saline‐exposed rats (b); however, cocaine‐exposed rats did not exhibit a statistically significant change in thigmotaxis in the OF relative to saline‐exposed rats (c). Cocaine‐exposed rats did not exhibit a statistically significant change in total arm entries in the elevated plus maze (EPM) relative to saline‐exposed rats (d), but spent more time exploring the open arms of the EPM than did saline‐exposed rats (e). *n* = 8/group for behavioral analyses. *p < 0.05 compared to saline. ^$^ *p* < 0.05 compared to cocaine baseline. ^#^ *p* < 0.05 compared to cocaine day 1. Error bars indicate *SEM*

Cocaine and saline groups did not differ in activity during the baseline session (Figure 2a). Cocaine‐exposed rats had significantly more activity than saline‐exposed rats on the first (*p* < 0.001) and last (*p* < 0.001) days of administration (Figure 2a). Rats sensitized to repeated cocaine injections; in the cocaine‐treated group, activity on the last day of administration was greater than activity on the first day of administration (*p* = 0.002) (Figure 2a).

### 3.2 Anxiety‐like and exploratory behavior

Rats exposed to cocaine followed by a 14‐day drug‐free period showed greater LMA in the OF than saline‐exposed rats (*p* < 0.01; Figure 2b), but cocaine‐ and saline‐exposed rats did not exhibit different degrees of thigmotaxis in this test (*p* = 0.478; Figure 2c). In the EPM, cocaine‐exposed rats showed a strong trend toward an increase in arm entries over saline‐exposed rats, although this effect did not reach significance (*p* = 0.054; Figure 2d), and spent a significantly greater amount of time in the open arms than did saline‐exposed rats (*p* = 0.024; Figure 2e). Cocaine exposure had no effect on distance traveled in the EPM (*p* = 0.11; data not shown).

### 3.3 LC *c‐fos* and TH

**Figure 3:**
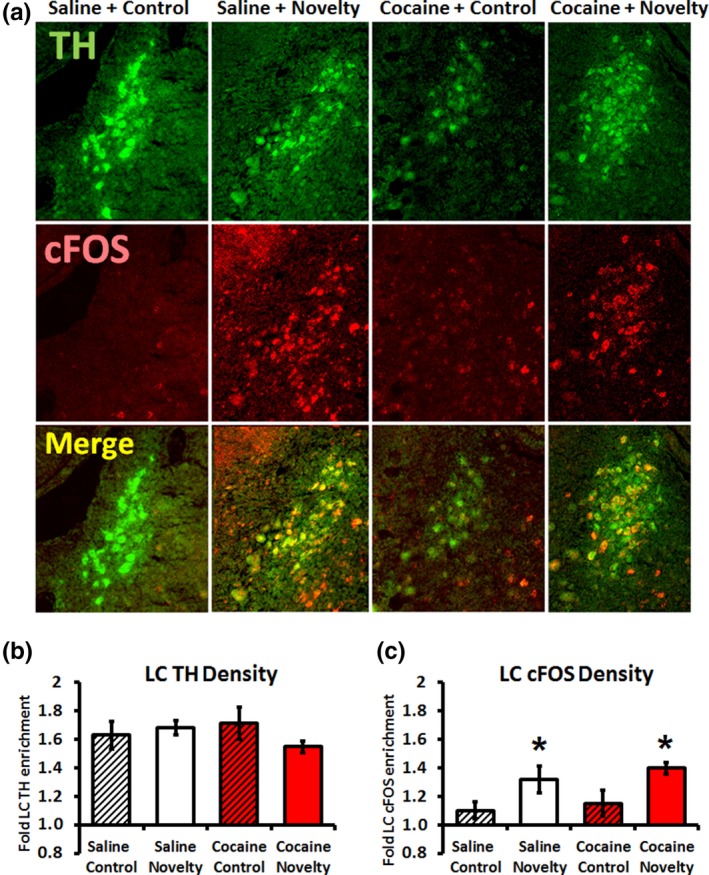
Novelty exposure induced locus coeruleus *c‐fos* expression. Labeling for tyrosine hydroxylase (TH) and *c‐fos* mRNA were apparent in locus coeruleus (LC) sections (a). Density of TH signal was not affected by cocaine administration, novelty exposure, or an interaction between the two (b). Expression of LC *c‐fos* mRNA was increased by novelty exposure, but affected neither by cocaine administration nor by an interaction between the two (c). *n* = 6 (saline control), 8 (saline novelty), 7 (cocaine control), and 5 (cocaine novelty) for fluorescence in situ hybridization immunofluorescence (FISH‐IF) analyses. *Main effect of novelty exposure, *p* < 0.01. Error bars indicate *SEM*

LC was readily identified by the presence of TH, and *c‐fos* expression was apparent within the LC (Figure 3a). Neither cocaine exposure (*F*(1, 22) = 0.067, *p* = 0.798), novelty exposure (*F*(1, 22) = 0.087, *p* = 0.770), nor an interaction between the two factors (*F*(1, 22) = 0.778, *p* = 0.387), affected LC TH signal intensity (Figure 3b). Novelty exposure increased LC *c‐fos* signal intensity (*F*(1, 22) = 9.047, *p* = 0.006), but there was neither a main effect of cocaine (*F*(1, 22) = 0.467, *p* = 0.501) nor an interaction between cocaine exposure and novelty (*F*(1, 22) = 0.282, *p* = 0.601) on this measure (Figure 3c).

### 3.4 Catecholamine measurements

Rats with a history of cocaine exposure had decreased AMY DA (*F*(1, 21) = 5.012, *p* = 0.036) but did not differ from control in NE concentration in any brain region analyzed (Table 1). Novelty exposure caused an increase in NE in the ACC (*F*(1, 27) = 5.130, *p* = 0.032) and a decrease in DA in the VTA (*F*(1, 21) = 5.012, *p* = 0.036; Table 1). No interactions between cocaine exposure and novelty were observed.

**Table 1:**
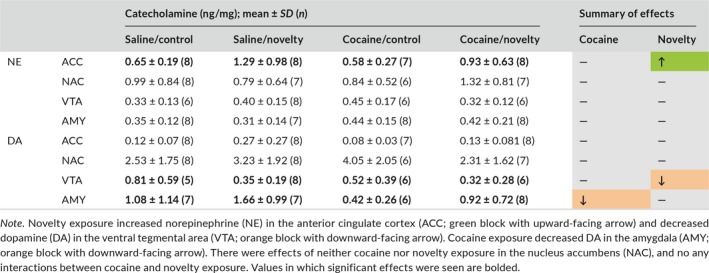
Cocaine and novelty exposure differentially affected brain catecholamines

### 3.5 Correlations between neurochemical and behavioral measurements

**Figure 4:**
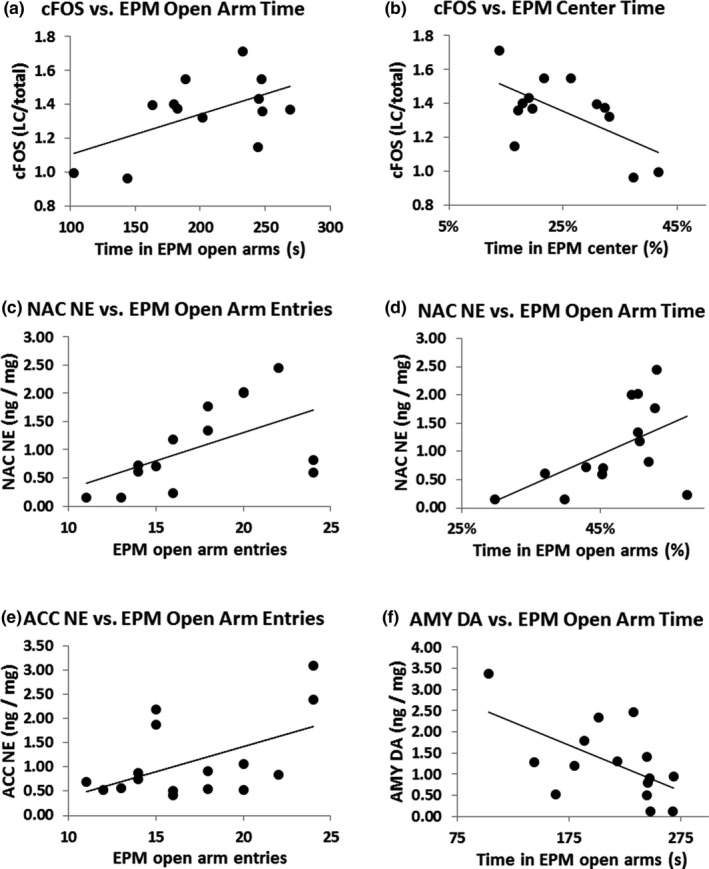
Significant correlations between neurobiological and behavioral variables. Expression of *c‐fos* mRNA in the locus coeruleus (LC) was positively correlated with time spent in the open arms of the elevated plus maze (EPM) (*r* = 0.635, *p* = 0.020; a). Expression of *c‐fos* mRNA was negatively correlated with time spent in the center of the EPM (*r* = −0.623, *p* = 0.023; b). Norepinephrine (NE) in the nucleus accumbens (NAC) was positively associated with increased open arm entries of the EPM (*r* = 0.540, *p* = 0.046; c). NE in the NAC was also positively correlated with increased percentage of time spent in the open arms of the EPM (*r* = 0.539, *p* = 0.048; d). NE in the anterior cingulate cortex (ACC) was positively associated with EPM open arm entries (*r* = 0.522, *p* = 0.038; e). Dopamine (DA) in the amygdala (AMY) was negatively associated with time spent in the open arms (*r* = −0.549, *p* = 0.034; f). Error bars indicate *SEM*

LC *c‐fos* was significantly positively correlated with time spent in the open arms of the EPM (*r* = 0.635, *p* = 0.020; Figure 4a) and negatively correlated with time spent in the center of the EPM (*r* = −0.623, *p* = 0.023; Figure 4b). NE in the NAC was correlated with increased open arm entries (*r* = 0.540, *p* = 0.046; Figure 4c) and increased percentage of time spent in the open arms (*r* = 0.539, *p* = 0.048; Figure 4d). NE in the ACC was correlated with EPM open arm entries (*r* = 0.522, *p* = 0.038; Figure 4e). DA in the AMY was negatively correlated with time spent in the open arms (*r* = −0.549, *p* = 0.034; Figure 4f).

## 4 DISCUSSION

This study confirmed our previous findings (Lisieski & Perrine, 2017) that repeated cocaine exposure followed by an extended drug‐free period caused a disinhibited, hyperexploratory phenotype in the OF and EPM tests. As in our previous study, cocaine exposure increased locomotor activity in the OF and open arm exploration in the EPM. In this study, cocaine exposure did not significantly increase thigmotaxis in the OF or locomotor activity in the EPM, which could be due to a dependence of these effects on the specific testing context, or an effect of the order of testing (as the EPM was always done after the OF); however, given the general concordance of these behavior effects with previous findings, these considerations do not strongly affect our interpretation of the data. This study also extended our previous results to show that while cocaine exposure dysregulated AMY DA, it did not increase novelty‐induced LC *c‐fos* expression or NE in the projection regions analyzed. Although there were no group‐wise effects of previous cocaine exposure on LC *c‐fos* expression or NE concentrations in this study, novelty exposure significantly increased NE in the ACC, and increased exploration of anxiogenic areas of the EPM was correlated with LC *c‐fos* expression and NE in the ACC and NAC. This pattern of associations suggests that changes in NE function may be related to disinhibited exploratory phenotypes such as those generated by repeated stimulant administration (Lisieski & Perrine, 2017; Mantsch et al., 2008; Olausson et al., 2000).

Our findings that AMY DA was decreased by exposure to binge‐pattern cocaine, and that low AMY DA concentration was associated with disinhibited exploration of the EPM, suggest that AMY DA dysregulation is important in the disinhibited phenotype seen following repeated cocaine administration and withdrawal. This is consistent with previous work which has found that DA depletion in the AMY increases exploration of the EPM open arms (Sullivan, Duchesne, Hussain, Waldron, & Laplante, 2009); however, that study found this effect was sexually dimorphic, present only in males. This raises the question of how the presently reported effect of cocaine exposure on AMY DA and its association with exploratory behavior are modulated by sex. Sexual dimorphism in AMY response to cocaine may be clinically relevant, as AMY involvement in some addiction‐relevant behaviors, such as cocaine cue response (Kilts, Gross, Ely, & Drexler, 2004), differs between men and women. Future studies are needed to address this possibility.

We found that ACC and NAC NE concentrations were correlated with exploration of the open arms of the EPM. ACC is densely innervated by LC and sends reciprocal connections to LC neurons; interactions between the ACC and LC appear to function as a switch between task‐focused and exploratory attentional states in rats (Aston‐Jones & Cohen, 2005; Kane et al., 2017). Our finding that NE ACC is positively associated with disinhibited exploration of the EPM is consistent with the idea that ACC–LC interplay regulates exploratory behavior. On the other hand, the NAC receives NE input from the primarily sensory nucleus tractus solitarius (NTS; Delfs, Zhu, Druhan, & Aston‐Jones, 1998). We did not make measurements in the NTS in this study, but the association between NAC NE release and disinhibited exploratory behavior in the EPM raises the possibility that some aspects of NTS physiology and signaling mediate exploratory behavior and may be dysregulated following cocaine. Previous findings from other laboratories show that NTS plays roles in modulating memory for affectively salient events such as morphine reward (Gonzalez‐Cuello et al., 2010; Olson et al., 2006) and aversive shocks (Kerfoot & Williams, 2011; Williams, Men, & Clayton, 2000). Therefore, the effects of repeated cocaine exposure on NTS physiology and its relationship to behavior may be fruitful targets for future study.

This study provides insight into relationships among LC activation, projection region catecholamine content, and exploratory behaviors that are disrupted following repeated cocaine exposure; however, it has some limitations. First, only male animals were used in order to simplify the interpretation of this study's results and because previous research on SPS and cocaine sensitization on which this study was based has largely been conducted using male animals. However, studies demonstrating sex differences in response to SPS (Keller, Schreiber, Staib, & Knox, 2015) underscore the importance of including female subjects in future studies. In this study, rats were sacrificed at a single time point following behavioral testing; therefore, it may be the case that some transient changes in catecholamines were not captured. In addition, technical limitations of our HPLC method precluded the measurement of low‐abundance compounds in our samples, so we did not measure neurotransmitter metabolites, and our use of sonicated tissue means that the levels measured reflect only whole‐tissue catecholamine levels, not catecholamine activity per se.

Further work is necessary to clarify the role of LC activity in neuroadaptations to cocaine exposure. Notably, while this study focused on changes in induced LC activation and NE in projection regions, other factors involved in noradrenergic neurotransmission may be affected by cocaine exposure. In the DA system, cocaine causes long‐term adaptations in multiple processes including tonic and phasic DA signaling, feedback inhibition, DA reuptake, potency of cocaine binding to DAT (Henry, Hu, & White, 1998), and induced plasticity in glutamatergic synapses onto DA cells in the VTA and its targets (van Huijstee & Mansvelder, 2015). Similarly complex changes that are yet to be explored may occur in the NE system following repeated exposure to cocaine and other stimulants. Given the roles of NE in modulating psychological processes disrupted in long‐term cocaine users such as sustained attention, working memory, and impulsivity (Potvin, Stavro, Rizkallah, & Pelletier, 2014), further study of cocaine‐induced adaptations in the LC as well as its cortical and limbic efferents may be vital in elucidating the biological basis of stimulant use disorders and their consequences. In addition, while we showed that LC *c‐fos* expression and regional NE concentrations are positively correlated with exploration of the anxiogenic open arms of the EPM, and that AMY DA is negatively correlated with this behavior, these data do not demonstrate causality. We hypothesize that high LC reactivity drove disinhibited exploratory behavior in our rats; indeed, this interpretation of the present findings is consistent with previous literature showing the LC (particularly in concert with ACC) promotes sustained exploration in novel environments (Gompf et al., 2010). However, it is also possible that exposure to more anxiogenic areas of the EPM was driven by other systems that were altered following repeated cocaine administration such as the midbrain dopaminergic system or the hypothalamic stress response system (Mantsch, 2017), and this secondarily affected LC activity and NE concentrations. Finally, LC neurons are heterogeneous in their projection regions, patterns of activity, and collateralization (Uematsu, Tan, & Johansen, 2015). Our dual FISH‐IF method, while allowing us to sensitively and specifically quantify *c‐fos* induction in the LC, did not allow us to differentiate functionally or anatomically distinct subpopulations of TH‐positive LC cells, which may differ in how they respond to novelty and adapt following exposure to cocaine.

In conclusion, our findings confirm that exposure to novel environments increases LC activity and NE in the ACC, and that disinhibited exploration in novel environments is correlated with NE and DA in regions that modulate decision‐making, risk‐taking, and avoidant behavior. The activation of LC/noradrenergic system did not appear to be enhanced in rats previously exposed to cocaine, although exposure to cocaine followed by withdrawal dysregulated AMY DA and caused a long‐lasting change in exploratory behavior. Given the importance of the noradrenergic system in regulating behaviors disrupted by cocaine and the links among LC activation, NE, and disinhibited behavior shown here, further studies investigating the effects of cocaine on brain catecholamine systems and related behaviors will be important for understanding the long‐lasting effects of cocaine on brain function.

## CONFLICT OF INTEREST

None declared.
